# The "Adulteration of Liquors."

**Published:** 1869-04

**Authors:** 


### ARTICLE XI.

### *The "Adulteration" of Liquors.*

The making of intoxicating drinks is perhaps the oldest of the chemical arts. In the regions where juicy fruits grow spontaneously, the fact of alcoholic fermentation must have been observed by the aborigines in their earliest experiences; the natural production of alcohol was constantly going on, under favoring circumstances, and man was liable and prepared to make its discovery. The change in taste of the luscious fruit which dropped from the branches may have been the first suggestion: but when sweet juices came to be used as a beverage the spontaneous turning of them into wine could not have been overlooked. The antediluvians were without doubt skilled in the art. Using the knowledge which he had got from his fathers, Noah when he came out the ark, planted a vineyard and used his home-made wine intemperately. There are those who to this day find about the world the lamentable consequences of one of his debauches.

The origin of the word wine, which, up to the fourteenth century, was applied to all the known alcoholic beverages, is lost in the mists of antiquity, It is certainly a remarkable and significant fact, that this word has been known to all nationalities and in all ages, a distinction shared only by a few household words which indicate the closest domestic relations.

Towards the end of their career, the antediluvians were very wicked, and it cannot be doubted that drunkenness was one of their vices. As in all the succeeding ages, wine was the inspiration of poets and the solace of the despairing.

Then was also begun that still enduring conflict between those who represent wine as the best gift of heaven, and those who find it the veriest poison of poisons. There were bacchanals, and we seriously suggest that there were also teetotal societies.

Wine has been praised and cursed among all peoples and in all times; the vocabularies of all languages have been exhausted for such uses, now of the softly glowing and now of the bitter words. The conflict too is carried into commerce and even into science, for the common names, which designate the alcoholic compounds, have with them something of their partisan origin. Witness: alcohol (THE liquid) spirit, *eau de vie*, whiskey. Not even gold enters so much into the thoughts and speech of men. Alcohol pays national debts, furnishes the popular songs, and crowds our prisons. The whole world may be made to quake if anything befall its king.

Eight or ten years ago one Dr. Cox came out of Ohio proclaiming strychnine in whiskey and sulphuric acid in wine.

Every one was frightened; but it is a curious and notable fact that horror fell heaviest on those good people who never taste or handle. It has been intimated, however, in explanation, that some of the anti-bacchants having become persuaded that alcohol, *per se*, is not really a very virulent poison, still insisted that it ought to be, and rejoiced at the making it so with strychnine and oil of vitriol; the horror on this theory was therfore a pretence. Finally it transpired that the strychnine story was the invention of some newspaper wag, and that Cox's evidence of sulphuric acid in wine was a precipitate on the additon of chloride of barium. (The precipitate was of course tartrate of baryta; an evidence of pure wine rather than of sulphuric acid.)

The grand climax of interest in the exposure of adulterations in the *New York World* was reached when the reports on liquors appeared, People were calm as long as it was only such things as tea, coffee, coal and bread which were shown to be mixed with worthless or deleterious rubbish, but when it was shown that the toddy is sophisticated and adulterated—Oh! it was dreadful! The tremendous developments could not await the slow publication by mail, and in a few hours the telegraph flashed over the continent to the gulf, to the Pacific, and far into Her Majesty's Dominion the blood-curdling fact that TANNIN and FUSEL OIL had been found in the fashionable New York liquors. Every one was aroused to the importance of the momentous announcement. The respectable Metropolitan Board of Health took the matter into solemn consideration, it furnished texts for sermons, and for leaders in newspapers; fusel oil was on the lips of the good and bad. The teetotal reformers congratulate and praise the *World* for its noble work in their cause. Granted that Cox's strychnine and sulphuric acid were myths, it is now demonstrated beyond recall that tannin and fusel oil are bodily present in drink. Bywords, compliments, rhymes, and jests suitable for the occasion, were invented for the street, the saloon, the theatre, and especially the minstrel halls. And the excitement still will last for many a day. * * * * * * * * * * * *

We suppose this to be a reasonable view of the question of the "adulteration" of liquors. The active agent of liquor is alcohol, and preferably ethylic alcohol; it is the alcohol which stimulates, exhilarates or poisons, Whatever is good or bad about liquor there is nothing better or worse than the alcohol. Water is added to temper the strength, and custom has established a proportion of half-and-half. Beyond the alcohol and water a flavoring substance is added, ordinarily in the form of an essential oil or ether. This flavoring matter gives the distinctive character of the different liquors; in brandy it is œnanthic ether; in gin, oil of juniper; in rum, butyric ether; in whiskey, the oil of the grain, &c. The flavoring is similar in nature to that we have in fruits, in flowers, in spices, and in most articles which we eat. As to quantity by weight, these aromas are always insignificant, and if they have any physiological action it is through the imagination or the nervous system. The question of flavor is generally one of taste and not to be disputed. The popular notions of "Jersey lightning," "rotgut whiskey," "mixed liquors," and "adulteration" of liquors have very little foundation in fact or reason. What a strange notion it is that our ancestors had better wines and liquors than we! Some people seem to think that fusel oil is a modern invention of knaves, which is used to "adulterate" liquor, when all that is ever done about it is to extract it.

"Will the coming man drink wine?" is asked, and we confidently answer nay, for our chemistry points to something better. Wine-making seems unreasonable to the scientific epicure of the nineteenth century. The tendency of modern thought and practice is toward the lopping off of the redundancies, crudities and impurities. We look more for the essences of things. The physicians who still administer burnt sponge and talk about the terrene virtue of mineral waters ought to laughed at. Let the temperance reformer find his adversary in alcohol only, regardless of what he is called, and utter no more nonsense about adulterated and poisoned liquors. Let the physician study the chemistry of liquors and find out how, all his life, he has humbugged himself as well as his patients. The coming physician will find in his materia medica only alcohol and certain flavors which will be more delightful than anything which we now can dream of.—*Chemical News.*

### MONTHLY SUMMARY.

